# Supplementary material for: Time‑resolved multi-omic analysis of paclitaxel exposure in human iPSC‑derived sensory neurons unveils mechanisms of chemotherapy‑induced peripheral neuropathy
Source: Cell Death Dis. 2026 Feb 10;17(1):211. doi: 10.1038/s41419-026-08445-2 (PMC12921266; doi:10.1038/s41419-026-08445-2)
Supplement: Supplementary file 15 — Dataset 1. [file 41419_2026_8445_MOESM15_ESM.zip › CDD_Open_Data/04_RNA_Seq/Timeline_panel_plots_etc_2023-01-23_1609.zip!/Panel_Plots_etc/Evidence_Plots/72h/evidence_plot_standard contrasts_msigdb_reactome_72h_PTX_vs_DMSO_ID0_M26917_REACTOME_RAF_INDEPENDENT_MAPK1_3_ACTIVATION.pdf]

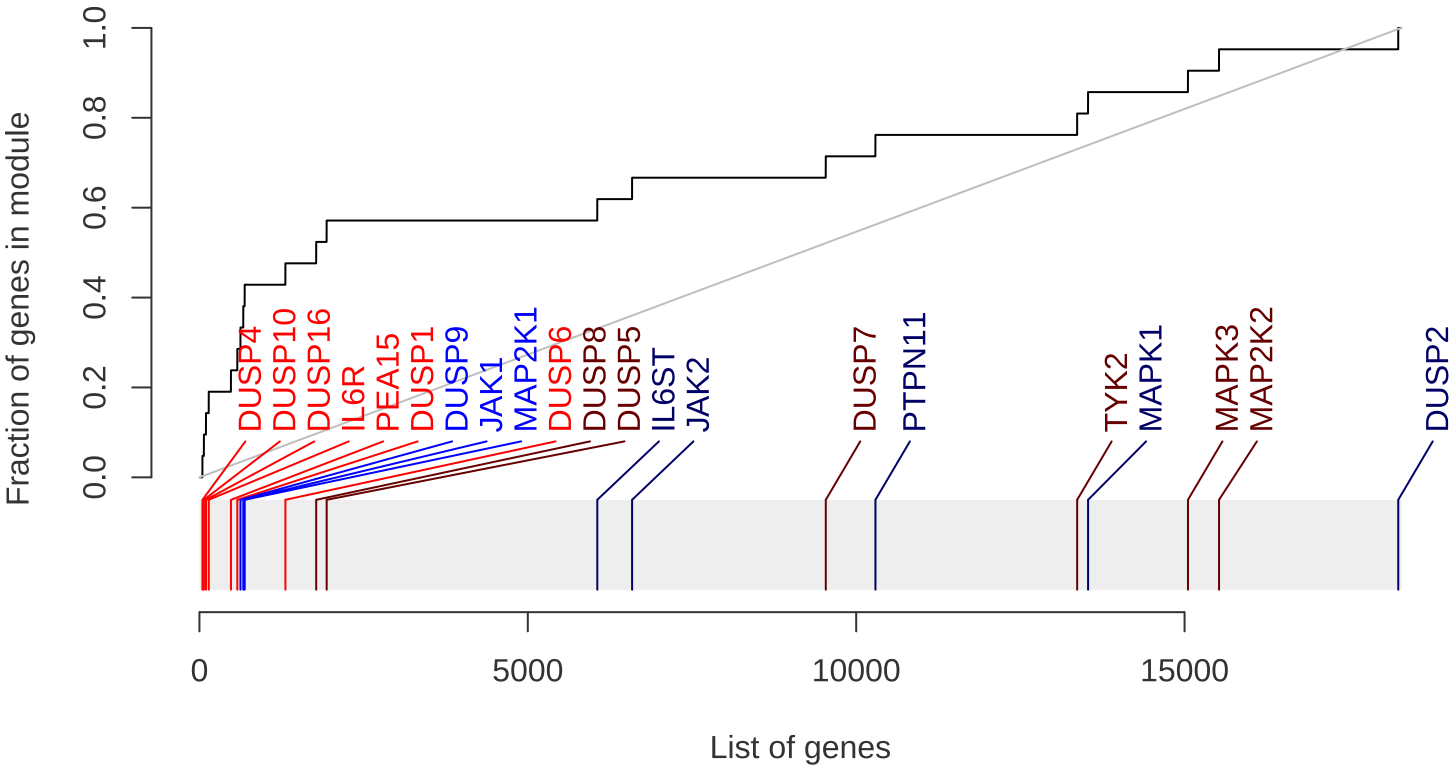
Fraction of genes in module
0.0
0.2
0.4
0.6
0.8
1.0
DUSP4
DUSP10
DUSP16
IL6R
PEA15
DUSP1
DUSP9
JAK1
MAP2K1
DUSP6
DUSP8
DUSP5
IL6ST
JAK2
DUSP7
PTPN11
TYK2
MAPK1
MAPK3
MAP2K2
DUSP2
0
5000
10000
15000
List of genes
